# The Arp2/3 complex is crucial for colonisation of the mouse skin by melanoblasts

**DOI:** 10.1242/dev.194555

**Published:** 2020-11-15

**Authors:** Vassilis Papalazarou, Karthic Swaminathan, Farah Jaber-Hijazi, Heather Spence, Ines Lahmann, Colin Nixon, Manuel Salmeron-Sanchez, Hans-Henning Arnold, Klemens Rottner, Laura M. Machesky

**Affiliations:** 1 CRUK Beatson Institute, Garscube Estate, Switchback Road, Bearsden, Glasgow G61 1BD, UK; 2 Institute of Cancer Sciences, University of Glasgow, Garscube Campus, Switchback Road, Bearsden, Glasgow G61 1QH, UK; 3 Centre for the Cellular Microenvironment, University of Glasgow, Glasgow G12 8LT, UK; 4 Cell and Molecular Biology, Institute of Biochemistry and Biotechnology, Technische Universität Braunschweig, Spielmannstrasse 7, 38106 Braunschweig, Germany; 5 Division of Molecular Cell Biology, Zoological Institute, Technische Universität Braunschweig, Spielmannstrasse 7, 38106 Braunschweig, Germany; 6 Department of Cell Biology, Helmholtz Centre for Infection Research, Inhoffenstrasse 7, 38124 Braunschweig, Germany

**Keywords:** Arp2/3, Melanoblasts, Migration, Actin cytoskeleton, Skin pigmentation

## Abstract

The Arp2/3 complex is essential for the assembly of branched filamentous actin, but its role in physiology and development is surprisingly little understood. Melanoblasts deriving from the neural crest migrate along the developing embryo and traverse the dermis to reach the epidermis, colonising the skin and eventually homing within the hair follicles. We have previously established that Rac1 and Cdc42 direct melanoblast migration *in vivo*. We hypothesised that the Arp2/3 complex might be the main downstream effector of these small GTPases. Arp3 depletion in the melanocyte lineage results in severe pigmentation defects in dorsal and ventral regions of the mouse skin. Arp3 null melanoblasts demonstrate proliferation and migration defects and fail to elongate as their wild-type counterparts. Conditional deletion of Arp3 in primary melanocytes causes improper proliferation, spreading, migration and adhesion to extracellular matrix. Collectively, our results suggest that the Arp2/3 complex is absolutely indispensable in the melanocyte lineage in mouse development, and indicate a significant role in developmental processes that require tight regulation of actin-mediated motility.

## Abstract (precis)

**Summary:** This study demonstrates that Arp2/3-dependent branched actin assembly is indispensable for melanoblasts populating the skin of the developing mouse embryo.

## INTRODUCTION

Tissue morphogenesis is in balance with cell migration. During development, cell movements are tightly regulated and highly coordinated in space and time. Melanocytes, the melanin producing cells that mainly reside in the bulge of hair follicles in adults, are derived from neural crest cells (Petit and Larue, 2016). In mice, neural crest cells start to migrate from the neural tube around embryonic day (E) 8.5 and the ones that follow the dorsolateral pathway give rise to melanoblasts, the embryonic precursors of melanocytes. From E9.0-E15.5, the melanoblast journey begins from the dorsolateral axis towards the developing dermis and involves crossing the basal layer to colonize the epidermis (from E11.5) and reach the first primary hair follicles (from E15.5) (Luciani et al., 2011). Specifically, between E8.5 and E10.5, the migration of melanoblasts is dorsolateral, whereas after E10.5 they migrate ventrally towards the face, ventral abdomen and the developing limbs. Around E13.5 they also migrate upward through the dermis and into the epidermis, crossing the basement membrane where they reside in hair follicles (Mayer, 1973; Thomas and Erickson, 2008).

Studies using immunostained tissue sections and live *ex vivo* imaging of mouse embryonic skin have highlighted that melanoblasts extend short protrusions and long pseudopods for migration. These protrusions are actin-rich and positively regulated by signalling through small GTPases and actin bundling proteins, including fascin (Ma et al., 2013). We have previously demonstrated that small GTPases Rac and Cdc42, but not RhoA, are important for melanoblast migration (Li et al., 2011; Woodham et al., 2017). Mice on a C57BL/6 background lacking Rac1 or Cdc42 in the melanocyte lineage showed a paucity of melanoblasts in the most distally located regions of migration, leading to lighter paws and tail tips and the formation of white belly spots. Furthermore, mice lacking P-Rex1, a Rac-specific GEF, exhibit similar pigmentation defects, highlighting the role of Rac signalling in melanoblast migration (Lindsay et al., 2011). Rac1 signals to the Arp2/3 (Actr2/3) complex via Scar (WAVE; Wasf1) to drive pseudopod extension, so it was expected that Rac1 nulls would not form normal protrusions. However, contrary to Rac1 knockouts, Cdc42 null melanoblasts were elongated, displaying dynamic pseudopods. Cdc42 is more complex, signalling via N-WASP (Wasl) and other effectors to coordinate the polarity of migration. Cdc42 null melanoblasts formed protrusions, but were unable to efficiently coordinate their adhesion and contractility machineries to use their protrusions for migration (Woodham et al., 2017). Thus, deciphering the signalling nodes that drive pseudopod formation and cellular motility are of paramount importance to understand melanoblast migration.

The Arp2/3 complex was initially identified in *Acanthamoeba* (Machesky et al., 1994; Mullins et al., 1997) and as a factor required for actin nucleation from the tails of *Listeria monocytogenes* pathogenic bacteria (Welch et al., 1997). The Arp2/3 complex is a major downstream target of Rac1, generating dendritic branches of actin filaments at cytoplasm-membrane interfaces. The Arp2/3 complex is organised around two central proteins, the actin-related proteins (Arp2 and Arp3) that interact with five different subunits (ArpC1-5). The Arp2/3 complex initiates actin polymerisation and is crucial for formation of membrane protrusions, such as pseudopods. Specifically, the ArpC1-5 subunits provide a framework for positioning the Arp2/3 subunits at the pointed ends of actin filaments and, as a result, the Arp2/3 complex initiates daughter filament branching at an ∼70° angle (Pollard, 1986, 2007). However, the study of the roles of the Arp2/3 complex has been challenged by the lethality of genetic disruptions of the complex from yeast to mouse model organisms (Schwob and Martin, 1992; Vauti et al., 2007; Yae et al., 2006).

Recent advancements in gene expression targeting tools suggested that loss of Arp2/3 complex abolishes formation of lamellipodial protrusions affecting cell spreading (Suraneni et al., 2012; Wu et al., 2012). The effects on cell migration appear to be influenced by the context of the study and the microenvironment. For example, ArpC3 knockout caused impaired chemotaxis towards EGF gradients (Suraneni et al., 2012), whereas combined ArpC2 and Arp2 depletion or chemical inhibition of the complex did not affect cell migration along PDGF gradients when cells were assayed under flow (Wu et al., 2012). Other observations have linked the Arp2/3 complex to focal adhesion organisation and dynamics (Wu et al., 2012), formation and maintenance of monolayers (Abu Taha et al., 2014), engulfment in macrophages (May et al., 2000) and membrane fusion (Sumida and Yamada, 2015). Interestingly, Arp2/3 loss has also been correlated with an NFκB-dependent induction of a senescence-associated secretory phenotype, suggesting that manipulation of the Arp2/3 complex during pathophysiology could confer non-autonomous effects that potentially influence cells and their environment in a systematic way (Wu et al., 2013).

Furthermore, mice with knockout of the ArpC4 subunit in keratinocytes [Arpc4^f/f^::K14-Cre(neo)] displayed skin abnormalities at birth that resulted in a psoriasis-like disease characterised by hyperactivation of the transcription factor Nrf2 (Nfe2l2). The authors suggested that the Arp2/3 complex is important for Nrf2 association with branched actin and, upon loss of the complex, Nrf2 accumulates in the nucleus (van der Kammen et al., 2017). ArpC3 deletion in the epidermis also using K14-Cre resulted in neonatal lethality and epidermal permeability barrier defects (Zhou et al., 2013). Zhou et al. concluded that Arp2/3 was important for epidermal tight junction formation and terminal differentiation, but surprisingly not required for cell-cell or cell-basement membrane adhesion (Zhou et al., 2013). Other minor phenotypes upon loss of ArpC3 have been reported in the small intestine (Zhou et al., 2015) and the central nervous system (Kim et al., 2013; Zuchero et al., 2015). However, whether the Arp2/3 complex affects the migration patterns that are crucial for tissue morphogenesis and development is still unknown.

Here, we sought to investigate the extent of the involvement of Arp2/3 complex in the melanoblast journey from the neural tube through the dermis and epidermis and into the hair follicles during mouse development. We demonstrate that mice depleted of Arp3 in the melanocyte lineage have a remarkable spotted coat colour phenotype characterised by large unpigmented dorsal and ventral areas and a paucity of melanocytes in those areas devoid of pigment. Arp3-depleted melanoblasts failed to colonize the epidermis from E13.5 and did not form pseudopods. Melanocytes lacking Arp3 displayed proliferation, adhesion and migration defects. Thus, we propose that the Arp2/3 complex is crucial for efficient melanoblast colonisation of the mouse skin.

## RESULTS

### Loss of Arp3 in the melanocyte lineage causes coat colour defects in mice

**Fig. 1.**
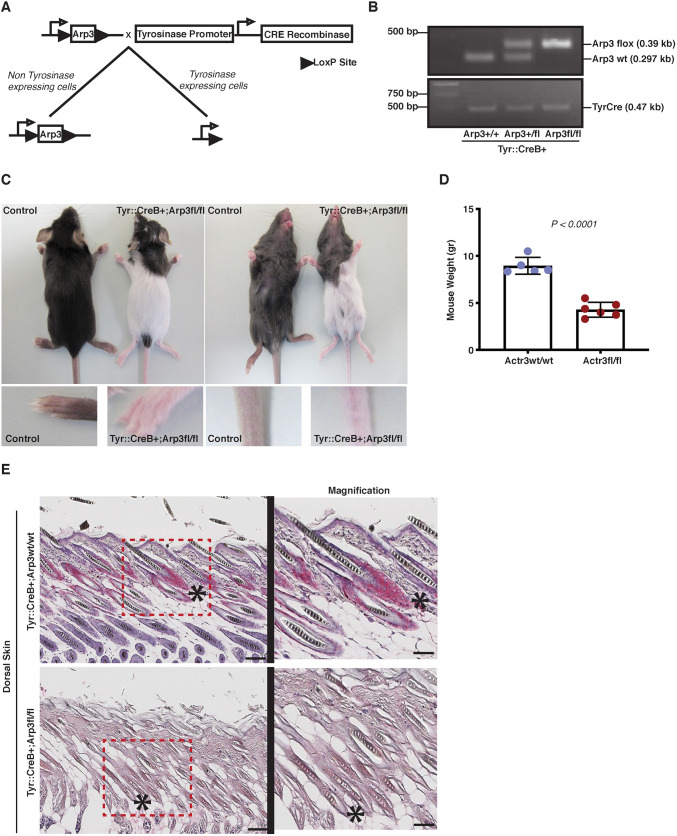
**Arp3 fl/fl Tyr::Cre^+^ mice display severe coat pigmentation defects.** (A) Schematic depicting gene targeting strategy for generating Arp3 (Arp3) fl/fl Tyr::Cre^+^ mice. Tyr::CreB expression mediates Arp3 excision in the melanocytic lineage. (B) Representative genotyping assay: Arp3 wt/wt Tyr::Cre^+^ (wild-type, left), Arp3 wt/fl Tyr::Cre^+^ (heterozygous, middle) and Arp3 fl/fl Tyr::Cre^+^ (homozygous, right). (C) Top: coat colour of P19 Arp3 fl/fl Tyr::Cre^+^ mouse with control (Arp3 wt/wt) littermate. Bottom: forelimbs and tails of mice from top panels. (D) Quantification of weight of Arp3 fl/fl Tyr::Cre^+^ mice and control littermates at P14. (E) Left: immunohistochemistry of dorsal skin sections of P14 control and Arp3 fl/fl Tyr::Cre^+^ mice showing S100 (red). Asterisks indicate hair follicles. Right: magnification of regions indicated by red box. Scale bars: 100 μm.

To assess the role of the Arp2/3 complex in melanoblast migration *in vivo*, we crossed mice carrying a floxed allele of Arp3 with mice expressing Cre recombinase under the control of the tyrosinase promoter (Tyr::Cre) (Delmas et al., 2003) in a C57BL6/J background (Fig. 1A,B, Fig. S1A). Arp3 fl/fl Tyr::Cre^+^ mice (*n*=16) exhibited a distinctive coat colour phenotype with large white patches along the dorsal and ventral areas of the mouse skin (Fig. 1C). Notably, coat pigmentation of Arp3 fl/fl Tyr::Cre^+^ mice displayed a non-regular phenotype, with the presence of pigmented spots most commonly in cranial and bottom caudal areas (Fig. S1B,C). Furthermore, limbs and tails in all Arp3 fl/fl Tyr::Cre^+^ mice were hypopigmented (Fig. 1C). In addition, Arp3 fl/fl Tyr::Cre^+^ mice were runty, shaky and smaller than littermates as evidenced by their reduced weight compared with littermates (Fig. 1D), phenotypes that can also be partially attributed to partial Cre transgene activity in the brain and peripheral nerves as previously reported (Delmas et al., 2003; Li et al., 2011).

Histological analysis using the melanocyte marker S100 of postnatal day (P) 14 control skin or skin from black areas of Arp3 fl/fl Tyr::Cre^+^ mice demonstrated the presence of melanocytes in the hair follicles, whereas melanocytes were essentially absent from white skin areas of Arp3 fl/fl Tyr::Cre^+^ mice (Fig. 1E). Immunofluorescence analysis on black areas of the skin of P14 Arp3 fl/fl Tyr::Cre^+^ mice suggests that DCT-positive melanocytes are not expressing Arp3 (Fig. S1D) or ArpC2 (p34) (Fig. S1E). In white patches of skin from Arp3 fl/fl Tyr::Cre^+^ mice, however, a few melanocytes were detected by DCT immunofluorescence that also appeared negative for Arp3 or ArpC2, as expected (Fig. S1D,E). In summary, loss of Arp3 from the melanocyte lineage causes a severe pigmentation defect, resulting in large areas of unpigmented fur that is largely devoid of melanocytes. Thus, Arp3 appears to be required for a normal melanocyte population in the skin of mice.

### Arp3 null melanoblasts fail to properly populate the developing mouse embryo

**Fig. 2.**
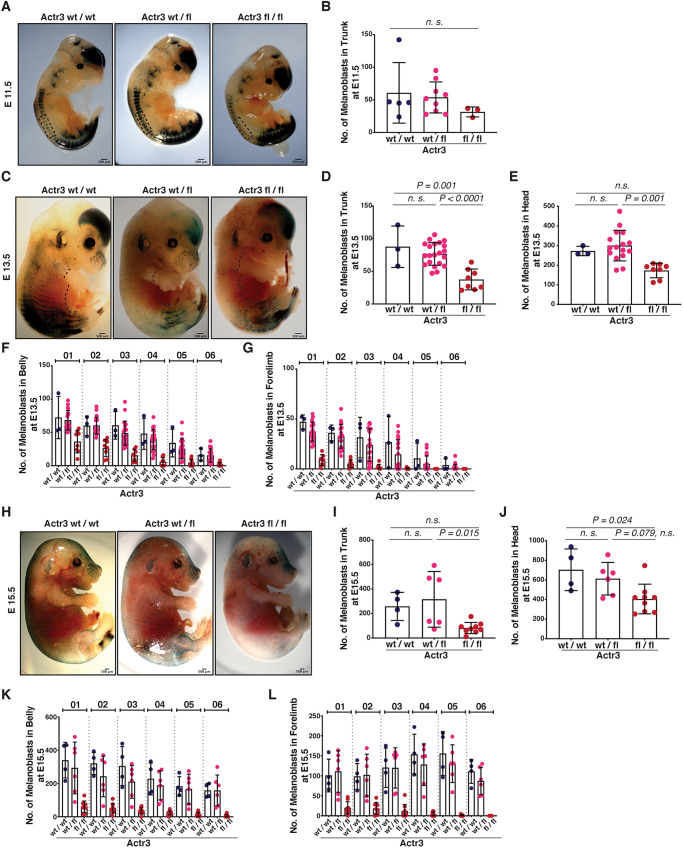
**Loss of Arp3 alters melanoblast number and distribution from E13.5.** (A-L) Embryos are DCT::*lacZ*; Tyr::Cre^+^ and either Arp3 wt/wt (‘wt’); Arp3 wt/fl (‘wt/fl’) or Arp3 fl/fl (‘fl’). (A) β-Galactosidase-stained whole-mount wt (left), wt/fl (middle) and fl (right) embryos at E11.5. (B) Number of melanoblasts in the trunk of embryos from A. Each dot represents an embryo. Data are mean±s.d. from four litters. Statistical significance was assessed using a Kruskal–Wallis with Dunn's multiple-comparisons test on *n*=5 wt, *n*=9 wt/fl and *n*=3 fl embryos. (C) β-Galactosidase-stained whole-mount wt (left), wt/fl (middle) and fl (right) embryos at E13.5. Black dotted lines indicate ventral migratory fronts. (D,E) Number of melanoblasts in the trunk (D) and the head (E) of embryos from C. Each dot represents an embryo. Data are mean±s.d. from four litters. Statistical significance was assessed by one-way ANOVA on *n*=3 wt, *n*=21 (D) or 16 (E) wt/fl and *n*=8 fl embryos. (F,G) Number of melanoblasts migrating in the belly (F) and the forelimb (G) of embryos from C. Each dot represents an embryo. Data are mean±s.d. from four litters. (H) β-Galactosidase-stained whole-mount wt (left), wt/fl (middle) and fl (right) embryos at E15.5. (I,J) Number of melanoblasts in the trunk (I) and the head (J) of embryos from H. Each dot represents an embryo. Data are mean±s.d. from three litters. Statistical significance was assessed by one-way ANOVA on *n*=4 wt, *n*=6 wt/fl and *n*=9 fl embryos. (K,L) Number of melanoblasts migrating in the belly (K) and the forelimb (L) of embryos from H. Each dot represents an embryo. Data are mean±s.d. from three litters. In F,G,K,L, 01-06 represent boxes of equal size spanning either the dorsal (1) to ventral (6) axis (F,K) or the top (1) to the distal tip (6) (G,L) as defined in Fig. S2. n.s., not significant. Scale bars: 500 μm.

Mouse melanoblasts emerge from the neural tube at ∼E10.5 and proliferate and migrate to populate the mouse skin by birth. Tyr::Cre^+^ is turned on at ∼E10.5, indicating the starting point of depletion of Arp3 from the emerging melanoblasts. Hence, we asked whether the observed coat colour defects upon Arp3 loss resulted from abnormal numbers and organisation of melanoblasts from E11.5 to E15.5. To address this, we used the reporter DCT*::lacZ* transgene (Mackenzie et al., 1997). Initially, at E11.5 when melanoblasts emerge from the neural tube and migrate dorsolaterally, the numbers of melanoblasts near the migration staging area of Arp3 fl/fl Tyr::Cre^+^ embryos were similar compared with control Arp3 wt/wt or heterozygous Arp3 wt/fl embryos (Fig. 2A,B). Qualitatively, the numbers of melanoblasts on the head/face area did not look different in mutant embryos at E11.5 (Fig. 2A,B). However, in E13.5 embryos, in which melanoblasts have started to expand and migrate ventrally (Fig. 2C), melanoblasts in control Arp3 wt/wt or wt/fl Tyr::Cre^+^ embryos populated midway across the embryo and part of the developing limbs, whereas Arp3 fl/fl Tyr::Cre^+^ embryos displayed a consistent deficit of melanoblasts in all areas (Fig. 2C), including the trunk (Fig. 2D, Fig. S2A), the head (Fig. 2E, Fig. S2B), the belly (Fig. 2F, Fig. S2C) and the forelimbs (Fig. 2G, Fig. S2D).

By E15.5, melanoblasts in control Arp3 wt/wt or wt/fl Tyr::Cre^+^ embryos have spread throughout the whole embryo including colonization of the developing limbs (Fig. 2H), whereas in Arp3 fl/fl Tyr::Cre^+^ embryos, melanoblasts were significantly less abundant in the trunk (Fig. 2I) and the head (Fig. 2J) and strikingly absent in the migratory pathways of the embryo belly (Fig. 2K) and forelimb (Fig. 2L). Thus, at early time points, the numbers and position of melanoblasts look relatively normal, but by E15.5, it is apparent that migration and proliferation are severely suppressed, most likely by the deletion of Arp3. This may be because of residual Arp3 at early time points, or lack of a requirement for Arp3 in early melanoblast specification.

### Arp3 null melanoblasts fail to properly form protrusions and to progress through the cell cycle in the developing embryo

**Fig. 3.**
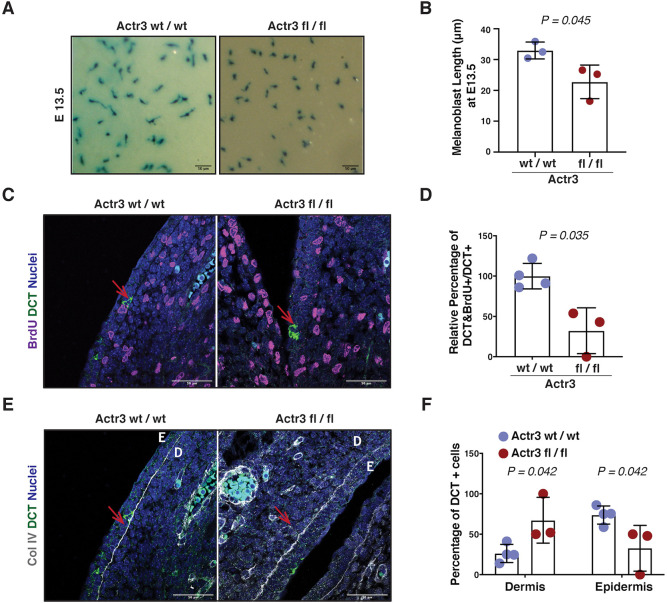
**Loss of Arp3 affects melanoblast protrusion, proliferation and epidermis homing from E13.5.** (A) High magnification images of belly melanoblasts of β-galactosidase-stained whole-mount DCT::*lacZ*; Tyr::Cre^+^ Arp3 wt/wt (left) or Arp3 fl/fl (right) embryos at E13.5. (B) Quantification of melanoblast length from A. Dots are mean of melanoblasts per embryo (at least 66 cells quantified per embryo). Data are mean±s.d. of *n*=3 wt/wt and *n*=3 fl/fl embryos. Statistical significance assessed by two-way unpaired *t*-test on mean values per embryo. (C) Representative images of E13.5 DCT::*lacZ*; Tyr::Cre^+^ Arp3 wt/wt (left) and Arp3 fl/fl (right) embryo transverse sections showing BrdU (magenta), DCT (green) and nuclei (blue). Red arrows indicate melanoblasts (DCT-positive). (D) Quantification of the DCT-positive cells that were also positive for BrdU from C. Each dot represents an embryo. Data are mean±s.d. from four litters. Statistical significance was assessed by two-tailed Welch's *t*-test. (E) Representative pictures of E13.5 DCT::*lacZ*; Tyr::Cre^+^ Arp3 wt/wt (left) and Arp3 fl/fl (right) embryo transverse sections showing collagen IV (Col IV; grey), DCT (green) and nuclei (blue). Red arrows indicate melanoblasts (DCT-positive). D, dermis; E, epidermis. (F) Quantification (percentage) of DCT-positive cells that reside in the dermis and the epidermis from E. Each dot represents an embryo. Data are mean±s.d. from four litters. Statistical significance was assessed by two-way unpaired *t*-test. Scale bars: 50 μm.

As defects in migration of Rac-depleted melanoblasts have been reported to correlate with the inability to extend pseudopods (Li et al., 2011), we assessed the cell shape of melanoblasts in E13.5 embryos upon Arp3 loss. Arp3-depleted melanoblasts were less elongated than melanoblasts of control Arp3 wt/wt Tyr::Cre^+^ embryos (Fig. 3A,B), in support of a key role for the Arp2/3 complex in long protrusions driving melanoblast migration.

We observed that Arp3-depleted melanoblasts were less abundant in trunk and head areas compared with control Arp3 wt/wt Tyr::Cre^+^ embryos at E13.5 and E15.5. This could also suggest a defect in cell cycle progression upon Arp3 loss in melanoblasts. As melanoblast numbers in Arp3 fl/fl Tyr::Cre^+^ embryos were already very low by E15.5, we investigated cell cycle progression of melanoblasts at E13.5. Specifically, BrdU was administered for 2 h and embryos were stained and quantified for BrdU incorporation in DCT-positive melanoblasts. We observed a significantly decreased incorporation of BrdU into Arp3-depleted melanoblasts (Fig. 3C,D, Fig. S3A). To assess whether Arp3 depletion could induce increased apoptosis in melanoblasts, we stained E13.5 embryos for cleaved caspase 3 as a marker of apoptotic cell death. Neither the control nor the Arp3-depleted melanoblasts showed cleaved caspase 3 expression (Fig. S3B,C). This does not exclude the possibility that Arp3-depleted melanocytes face survival challenges at this embryonic stage; however, our data suggest a delay in cell cycle progression, similar to the one observed in the Rac1 and Cdc42 knockout models (Li et al., 2011; Woodham et al., 2017).

To test whether eliminating a major pathway toward senescence in melanoblasts could overcome the survival challenges of Arp3 deletion and rescue the coat colour phenotype, we crossed Arp3 fl/fl Tyr::Cre^+^ mice with mice deficient in p16-Ink4a (Cdkn2a). However, Arp3 depletion in p16-Ink4a knockout melanocytes resulted in a similar defect in coat pigmentation in mice (Fig. S3D), indicating that senescence was not likely causing the growth arrest. We conclude that Arp3 depletion decreases proliferation of melanoblasts, impairing their population of the mouse skin during embryogenesis.

Furthermore, we observed a reduced number of Arp3-depleted melanoblasts crossing the basement membrane and reaching the epidermis at E13.5 (Fig. 3E,F). This could be the result of reduced migratory capacity of Arp3-depleted melanoblasts, but could also suggest a defect in the formation of efficient adhesions to the basement membrane that would allow them to traverse the basement membrane. Overall, loss of the Arp2/3 complex causes severe migratory as well as proliferation defects in the melanocyte lineage, which cannot be rescued by loss of p16-Ink4a.

### Arp3 loss affects proliferation and migration in primary cultured melanocytes

**Fig. 4.**
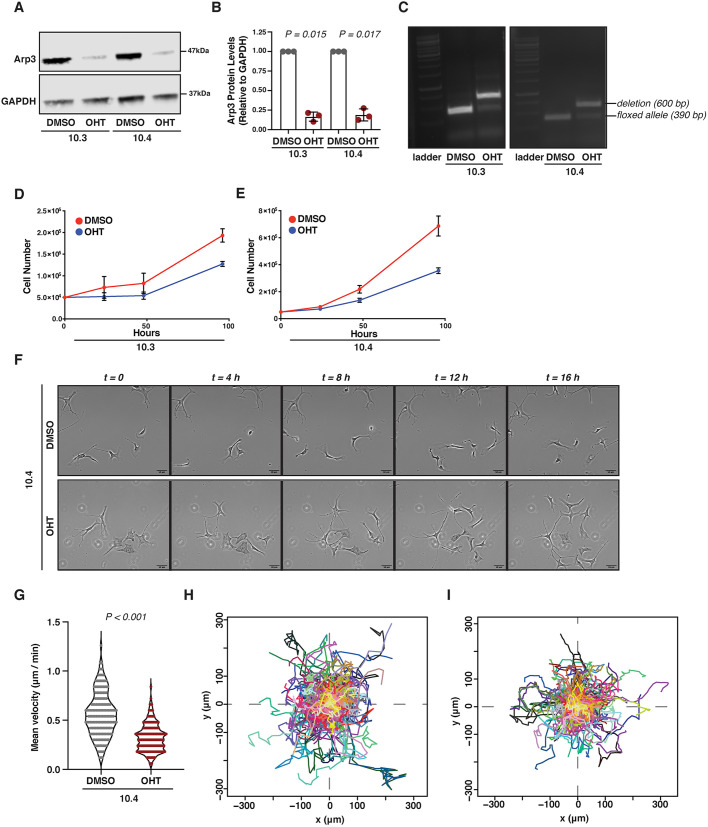
**Primary murine melanocytes depend on Arp3 for efficient proliferation and migration on fibronectin-coated surfaces.** (A) Immortalised primary melanocyte 10.3 and 10.4 lines were treated with OHT or DMSO for 7 days and immunoblotted for Arp3 and GAPDH (loading control). Pictures are representative from three independent experiments. (B) Densitometric quantification of protein in A. Data are mean±s.d. of three independent experiments. Statistical significance was assessed by two-tailed one-sample *t*-test on natural log-transformed values. (C) 10.3 and 10.4 primary melanocytes from A were assessed for the presence of the floxed Arp3 allele. Deletion was confirmed by a 600 bp-fragment band. Pictures are representative from three independent experiments. (D,E) Growth curves of 10.3 (D) and 10.4 (E) primary melanocytes from A monitored for 96 h following 7 days of OHT treatment. Data are mean±s.d. from three independent experiments. (F) 10.4 primary melanocytes treated either with DMSO (top) or OHT (bottom) for 7 days were cultured on fibronectin-coated surfaces for 16 h. (G) Cell speed from F. Data are median and interquartile range from *n*=170 DMSO- and *n*=196 OHT-treated cells from three independent experiments. Statistical significance was assessed using a Mann–Whitney *U*-test. (H,I) Tracks of cells (spider plots) of DMSO- (H) or OHT- (I) treated cells from F. Scale bars: 50 μm.

To further understand the role of the Arp2/3 complex in cell behaviour, we isolated and cultured immortalised primary melanocytes from P2 pups of Arp3 fl/fl; Rosa26::Cre-ER^T2^; Ink4a-Arf^−/−^ background to permit conditional deletion of Arp3 with the tamoxifen analogue, 4-hydroxytamoxifen (OHT) in culture. We confirmed Arp3 loss in two lines of primary cultured melanocytes, 10.3 and 10.4, following 7 days of OHT treatment (Fig. 4A-C), that was also followed by loss of ArpC2, suggesting disruption of the entire Arp2/3 complex (Fig. S4A). *Arp3* mRNA was also lowered in OHT-treated primary melanocytes but we saw no increase (or change) in the recently described *Arp3b* isoform (Abella et al., 2016) (Fig. S4B,C). We next sought to investigate whether loss of the Arp2/3 complex challenges the proliferative capacity of primary melanocytes *in vitro*. Therefore, we treated 10.3 and 10.4 cells with OHT for 7 days and then assessed their growth capacity over 96 h. Indeed, OHT-mediated depletion of *Arp3* impaired growth of both primary melanocyte cell lines (Fig. 4D,E), correlating with our *in vivo* observations. It is worth mentioning that loss of the Arp2/3 complex did not qualitatively affect pigmentation in primary melanocytes (Fig. S4D). This observation is in agreement with the pigmentation that we observed in the head area of Arp3 fl/fl Tyr::Cre^+^ mice, suggesting that even though proliferation and migration of melanocytic cells is severely impaired by loss of Arp3, if they can manage to populate hair follicles, they can still produce melanin and transfer it to the hair.

Control primary melanocytes appeared to form enlarged lamellipodial protrusions on fibronectin-coated surfaces and moved at an average speed of 0.53 μm/min. However, following deletion of *Arp3* (using OHT), cells lost their lamellipodial morphology, exhibiting thin protrusions and irregular or even rectangular shape (Fig. 4F). Arp2/3-depleted primary melanocytes migrated around 1.6× slower than controls, at a speed of 0.32 μm/min (Fig. 4G-I), in line with previous studies showing impairment but not total loss of migration in Arp2/3-depleted cells (Wu et al., 2012).

### The Arp2/3 complex is important for ECM adhesion and cell body integrity in primary melanocytes

Cell contacts with the extracellular matrix (ECM), known as focal adhesions, are important for proper cell spreading, proliferation and migration. Focal adhesions are not only major signalling hubs but constitute the main bidirectional ports of biomechanical cues between cells and the ECM. Both integrin activation and phosphorylation of signalling components of focal complexes, including FAK (Ptk2) or paxillin have been reported to be involved in Rac activation at the leading edge (Berrier et al., 2002; Kurokawa et al., 2004; Nayal et al., 2006; Sharma and Mayer, 2008). Rac-deficient fibroblasts have been reported to lack lamellipodia formation; however, these cells were able to spread, to form filopodia and to establish focal adhesions (Steffen et al., 2013). The Arp2/3 complex has been directly associated with components of focal adhesions including the proteins vinculin and FAK, promoting leading edge formation and cell migration (Chorev et al., 2014; DeMali et al., 2002; Serrels et al., 2007; Swaminathan et al., 2016). Therefore, we next sought to investigate focal adhesion formation in Arp2/3-deficient primary melanocytes.

**Fig. 5.**
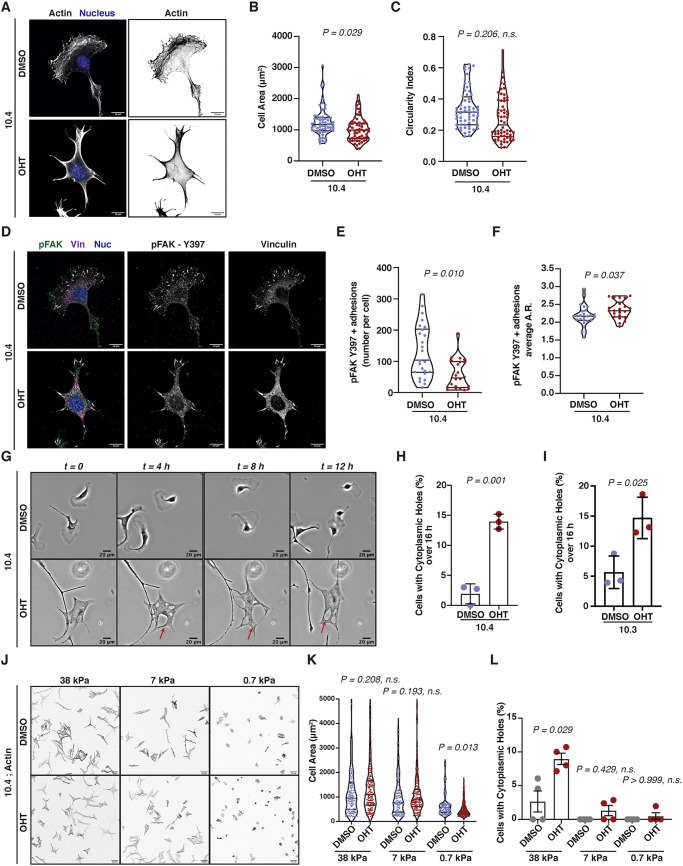
**Primary murine melanocytes depend on Arp3 for proper focal complex and membrane formation.** (A) Immunofluorescence of 10.4 immortalised primary melanocytes treated with DMSO or OHT for 7 days and plated on fibronectin-coated coverslips showing actin and nuclei. Right panels show actin channel only. (B,C) Quantification of shape of cells in A including cell area (μm^2^) (B) and circularity index (C). Data are median and interquartile range from *n*=60 DMSO- and *n*=61 OHT-treated cells. Cells were from three independent experiments. Statistical significance was assessed using a two-tailed Welch's *t*-test on mean values per experiment. (D) Immunofluorescence of 10.4 immortalised primary melanocytes treated with DMSO or OHT for 7 days and plated on fibronectin-coated coverslips showing pFAK-Y397 (green), vinculin (magenta) and nuclei (blue). Middle panels show p-FAK-Y397 channel. Right panels show vinculin channel. (E,F) Quantification of p-FAK-Y397-positive particles from D showing the number of focal adhesions per cell (E) and the average aspect ratio (A.R.) of focal adhesions per cell (F). Data are median and interquartile range from *n*=26 DMSO- and *n*=23 OHT-treated cells. Cells are from three independent experiments. Statistical significance was assessed using a Welch's *t*-test. (G) 10.4 primary melanocytes treated either with DMSO (top) or OHT (bottom) for 7 days were cultured on fibronectin-coated surfaces for 16 h. Representative images are shown at *t*=0, 4, 8 and 12 h. Red arrows indicate cell body holes. (H,I) Quantification of the percentage of 10.4 (H) and 10.3 (I) primary melanocytes showing cytoplasmic holes on fibronectin-coated surfaces over 16 h. Data are mean±s.d. from three independent experiments. Statistical significance was assessed using a two-tailed Welch's *t*-test. (J) Immunofluorescence of 10.4 immortalised primary melanocytes treated with DMSO or OHT for 7 days and plated on fibronectin-coated 38 kPa, 7 kPa and 0.7 kPa polyacrylamide hydrogels showing actin. (K) Quantification of cell area (μm^2^) from cells in J. Data are median and interquartile range from four hydrogels per stiffness within the same day. Statistical significance was assessed using a Kruskal–Wallis with Dunn's multiple-comparisons test on *n*=128 DMSO- and *n*=129 OHT-treated cells on 38 kPa; *n*=103 DMSO- and *n*=126 OHT-treated cells on 7 kPa; *n*=93 DMSO- and *n*=170 OHT-treated cells on 0.7 kPa. (L) Quantification of the percentage of 10.4 primary melanocytes showing cell body holes from J. Data are mean±s.e.m. from four hydrogels per stiffness within the same day. Statistical significance was assessed using two-tailed Mann–Whitney *U*-test. Scale bars: 10 μm (A,D); 20 μm (G); 50 μm (J).

Arp2/3-deleted primary melanocytes cultured on fibronectin-coated surfaces appeared smaller (median area of 996 μm^2^) than control cells (median area of 1170 μm^2^) but not significantly less circular (Fig. 5A-C, Fig. S5A,B). Cells were then stained for focal adhesion markers pFAK-Y397, vinculin (Fig. 5D) and p-paxillin-Y118 (Fig. S5E). Control cells had more pFAK-Y397-positive focal adhesions than *Arp3*-depleted primary melanocytes (Fig. 5E). Furthermore, upon loss of the Arp2/3 complex primary melanocytes formed longer pFAK-Y397 adhesions than control cells, as evidenced from an increased aspect ratio (AR) of the positive particles (Fig. 5F). Similar trends were also observed regarding vinculin- (Fig. S5C,D) and p-paxillin-Y118- (Fig. S5F,G) positive focal adhesions.

The focal adhesion phenotype upon Arp2/3 complex loss in primary melanocytes suggests that cells might have lost the potential for dynamic remodelling of their ECM contacts, which would explain their decreased capacity for migration *in vitro* as well as *in vivo*. Considering also the difficulty of Arp3-depleted melanoblasts crossing the basement membrane in the developing embryo, the Arp2/3 complex could be important for efficient cell-ECM contact dynamics in development. In addition, the trend for longer adhesions in *Arp3-*depleted melanocytes could also suggest increased membrane tension or an imbalance between contractile activity and cortical integrity or protrusive activity. Remarkably, we observed major cytoplasmic membrane holes during the migration of *Arp3*-depleted primary melanocytes on fibronectin-coated surfaces (Fig. 5G and Fig. S5H,I). Around 15% of *Arp3-*depleted primary melanocytes presented cytoplasmic holes at least once during16 h of migration on fibronectin-coated surfaces (Fig. 5H,I). We hypothesised that the excessive stiffness of the culture dish could impose a significant challenge for cells, enabling them to form some focal complexes that could direct cell spreading but at the cost of membrane holes and inability to form lamellipodia upon Arp2/3 loss. Therefore, we cultured primary melanocytes on fibronectin-coated hydrogels ranging from 0.7 to 38 kPa and assessed their spreading (Fig. 5J). Interestingly, although upon an ECM stiffness value >7 kPa, which represents stiff tissues such as skin epidermis (Butcher et al., 2009), *Arp3*-depleted cells appeared similar in size to control cells, on a soft 0.7 kPa ECM, a value closer to the stiffness of embryonic tissues (Lau et al., 2015), *Arp3*-depleted primary melanocytes appeared smaller than control cells (Fig. 5K). In the latter conditions, cells were also less prone to form holes (Fig. 5L). Taken together, these data suggest that loss of the Arp2/3 complex could be important for efficient cell-ECM contact formation as prerequisite of migration in the developing mouse embryo.

## DISCUSSION

Loss of Arp3 in the melanocyte lineage results in severe coat colour defects in mice and suggests essential roles for the Arp2/3 complex in melanoblast migration, progression through the cell cycle and crossing of the basement membrane in the developing skin, yielding important insights in the roles of the Arp2/3 complex in development. Loss of Arp3 in mice is embryonically lethal at the blastocyst stage, with E3.5 blastocysts failing also to develop *in vitro* (Vauti et al., 2007). This suggests that Arp3 has vital roles for early mouse development, in which tight regulation of cellular motility events is of paramount importance. Here, we show that the melanoblast lineage is highly dependent on Arp3 for population of the skin and hair follicles.

Arp2/3 is likely the main actin cytoskeletal target of Rac1 and Cdc42, which both have severe migration and proliferation defects when depleted from melanoblasts (Li et al., 2011; Woodham et al., 2017). However, the phenotype of loss of Rac1 or Cdc42 in melanoblasts was milder, as loss of pigmentation was mainly evident in the most distant sites of the melanoblast migratory path, i.e. the mouse belly (Li et al., 2011; Woodham et al., 2017). Both Rac1 and Cdc42 can interact with and activate the main nucleation promoting factors that then activate Arp2/3 – N-WASP and Scar (Insall and Machesky, 2009). Unlike Rac1 or Cdc42 removal, loss of Arp3 also inhibited melanoblast adhesion and crossing of the basement membrane and resulted in a severe depletion over most of the trunk skin of the mice, reflecting how fundamental its function is. This may reflect that in Rac1 knockouts there will be Cdc42 activity and likewise for Cdc42 knockouts, and that evidence suggests potential compensation of the WASP-family proteins N-WASP and Scar (Insall and Machesky, 2009; Veltman et al., 2012). The small areas on the head and rump of the mice that were populated with melanoblasts showed black pigmentation, indicating that even without Arp2/3, mature melanocytes can home to hair follicles and produce pigment. This finding warrants further investigation, as we were unable to culture these cells or to detect Arp3 in them *in situ* with antibody, but we could not absolutely prove that they contained no Arp2/3 complex. However, it is clear that Arp2/3 is a crucial driver of the melanoblast journey and is a key player in cell cycle progression downstream of Rac1 and Cdc42 during developmental expansion of the melanocyte lineage.

The Arp2/3 complex nucleates branched actin filament networks. Its activity is stimulated by nucleation promoting factors, including the Wiskott-Aldrich syndrome proteins (WASP and N-WASP), the WASP family verprolin-homologous protein (SCAR) and the WASP and SCAR homologue (WASH) complex. Previous evidence suggests that N-WASP- and WASH-dependent processes are dispensable for efficient melanoblast migration *in vivo* (Li et al., 2011; Tyrrell et al., 2016). Furthermore, loss of the Rho GTPase RhoA in the melanocyte lineage did not affect melanoblast migration or skin pigmentation (Woodham et al., 2017). As Arp2/3 is a downstream target of both Rac1 and Cdc42, taken together, these studies and our current data suggest that signalling via Rac1 to Arp2/3 and via Cdc42 to multiple effectors define the most crucial pathways (of those so far examined) among Rho GTPases and actin nucleation promoting proteins for melanoblast population of the skin. However, as the Arp2/3 complex is not a direct target of Rac1 or Cdc42, there must be a nucleation promoting factor mediating the signalling. We speculate that the Scar complex is the likely crucial link, at least for Rac1, but this awaits further experiments, such as knockout of a Scar complex subunit in melanoblasts.

White spotting mutants in mice arise due to improper melanoblast specification, survival and migration (Baxter et al., 2004). Interestingly, proliferation and migration defects have appeared to coincide in the melanocyte lineage *in vivo*, following genetic manipulation of motility proteins (Li et al., 2011; Lindsay et al., 2011; Ma et al., 2013; Woodham et al., 2017). During embryogenesis, melanoblasts initially emerge in the dermis, which has a unique ECM of collagen and proteoglycans at E10.5 in the head and cervical regions. By E11.5, melanoblasts appear in the trunk and tail and, after E13.5, melanoblasts cross the basement membrane and arrive in the epidermis. Arp3 deletion resulted in a range of pigmentation phenotypes, ranging from almost complete absence of pigmentation to major black spotted regions of the cranial and caudal areas both in dorsal and ventral skin. However, the most common phenotype was mice with only a pigmented patch in the head and one in the bottom trunk, a phenotype similar to ‘piebald type spotting’. This type of phenotype has been suggested to be mainly the result of lower melanoblast density in the areas of white spotting (Pavan and Tilghman, 1994; Yoshida et al., 1996). Although we observed relatively normal number and position of melanoblasts at an early stage (E11.5), where melanoblasts may still have residual Arp2/3 activity, by E13.5 and till E15.5, melanoblasts appeared reduced in number not only in distant areas such as the embryo belly and the developing front leg, but also in the upper trunk and the head. Melanocytes were largely absent from the white patches of skin in Arp3 flox mice, similar to what we observed for Rac1 or Cdc42 pathway mutants or fascin knockouts. The Arp3 flox phenotype was more severe than either Rac1 or Cdc42 loss, indicating that Arp2/3 is likely downstream of both GTPases and has a more fundamental role.

Conditional knockout of Arp3 in primary melanocytes ablated Arp2/3 complex function, causing loss of lamellipodia formation and the appearance of holes through the cell body. These phenotypes were associated with decreased proliferation and reduced migration on fibronectin-coated surfaces. Primary melanocytes showed only moderately reduced spreading area on fibronectin-coated glass, but they displayed a less rounded shape and numerous holes through the cell body, as if they lacked integrity to resist the stretching forces of spreading. However, on soft ECM, within the range that migratory melanoblasts sense *in vivo*, Arp3-depleted cells failed to spread as efficiently as the controls and showed fewer holes. Thus, loss of Arp2/3 likely weakens the cortical cytoskeleton, making the cell less robust against spreading forces. Previous studies implicated Arp2/3 in cortical actin assembly and integrity (e.g. Bovellan et al., 2014; Pavan and Tilghman, 1994; Yoshida et al., 1996), most recently in T-cells, in which a partial loss triggered blebbing and reduced cortical tension (Obeidy et al., 2020). In addition, the Arp2/3 complex directly or indirectly associates with components of focal complexes, such as vinculin or FAK, coupling substrate-sensing-dependent clustering of integrin receptors to actin polymerisation, membrane protrusion and cell motility (DeMali et al., 2002; Swaminathan et al., 2016). Therefore, apart from increased membrane tension or imbalanced actomyosin contractility, misregulated turnover of focal adhesions could be involved in the generation of the membrane holes observed. Interestingly, improper focal adhesion turnover has been shown to induce larger focal contacts and plasma membrane ruptures (Peacock et al., 2007). Elucidating the exact sequence of events in the integrin activation-focal adhesion-actin polymerisation axis and how they are involved in regulating membrane homeostasis will be of interest in the future. In addition, how focal adhesion disassembly factors traffic to focal contacts and the extent of the requirement for branched actin filaments are still not completely understood. Nevertheless, both the adhesion and the actin polymerisation machineries are strongly interconnected (Ciobanasu et al., 2012), and are not only crucial for transmission of biological signals but also highly important mechanical and structural regulators of cytoplasmic architectural homeostasis.

Our work suggests that loss of the Arp2/3 complex in primary melanocytes caused deregulation of focal contacts *in vitro* and migration across the basement membrane *in vivo*. Focal adhesions in Arp3 null cells appeared fewer in number but more elongated. Although the connection between branched actin and adhesion is still not well understood, focal adhesion proteins such as FAK can bind directly to the Arp2/3 complex, linking integrin signalling to the cytoskeletal machinery (Serrels et al., 2007). Furthermore, the interactions between FAK and the Arp2/3 complex have been reported to be essential for sensing ECM concentration during migration and haptotaxis (Swaminathan et al., 2016). Arp2/3 may also transiently bind to vinculin (DeMali et al., 2002). Interestingly, the integrin-activating adaptor protein kindlin-2 (Fermt2) interacts with paxillin to activate Rac1 and associates with the Arp2/3 complex to induce membrane protrusions (Böttcher et al., 2017). Local Arp2/3-dependent actin polymerisation may thus regulate efficient traction force application, buffering nascent adhesions from the mechanical effects of retrograde flow (Buck et al., 2017). Arp2/3-depleted fibroblasts failed to properly align focal adhesions (Wu et al., 2012). Collectively, Arp2/3-mediated formation of lamellipodia and associated retrograde actin flow appears to be essential for proper formation and alignment of focal adhesions contributing to directed migration both *in vitro* and *in vivo*. Interestingly, a similar cellular phenotype has also been observed in a podocyte-specific deletion of Arp3 (Schell et al., 2018).

In summary, melanoblasts rely on Arp2/3 to proliferate and migrate in embryo skin to eventually populate hair follicles and provide pigment in mice. Loss of Arp3 from the melanoblast lineage resulted in a more severe depletion of melanoblasts from the truncal skin of mice than previously reported for Rac1 and Cdc42 loss (Li et al., 2011; Woodham et al., 2017), suggesting a fundamental role for Arp3 not only in migration, but also developmental expansion in the skin. We show that Arp3-depleted melanocytes in culture have severe defects in cytoskeletal integrity and adhesion, likely playing a central role *in vivo* as melanoblasts migrate from the neural crest and cross barriers to reach their destinations in the skin and hair follicles.

## MATERIALS AND METHODS

### Generation of the mouse mutant carrying the conditional Arp3^loxP/loxP^ allele

The mouse Arp3 gene was modified by homologous recombination in *Escherichia coli* as described by Liu et al. (2003) and by conventional DNA technology as depicted in Fig. S1A. The gene locus was isolated from a 129s7 BAC library and then equipped with 2 loxP sites by PCR amplification within introns 1 and 3 to allow for deletion of Arp3 exons 2 and 3, respectively, by Cre recombinase. In addition, the PGK-neo and the diphtheria toxin (DTA) cassettes were introduced to the construct for positive and negative selection in embryonic stem cells (ESCs). An extra HindIII restriction site was also added downstream adjacent to the second loxP element to facilitate detection of the Arp3loxP allele. The linearised vector construct encompassing 12 exons of the Arp3 gene was electroporated into E14tg2A ESCs, which were then selected and screened for homologous recombination within the Arp3 locus. Several independently derived ESC clones bearing the correctly floxed mutant allele were aggregated with cd-1 morulae and the chimeric embryos were successfully transferred to foster mothers to obtain the corresponding mouse mutant lines. All of the DNA recombination and transgenic mouse work was performed at the Technische Universität Braunschweig, Germany. Mice were handled in accordance with the national animal welfare law and following instructions of local authorities. The derived mutant mouse strain was then shipped to the Beatson Institute, Glasgow, Scotland, where it was crossed with Tyr1 Cre mice (Delmas et al., 2003) in C57BL6/J background to obtain the specific Arp3 deletion in melanocytes.

### Transgenic mice and genotyping

Mice were maintained in the Animal House of the Beatson Institute according to UK Home Office regulations and in compliance with EU Directive 2010/63 and the UK Animals (Scientific Procedures) Act 1986. All protocols and experiments were previously approved by the Animal Welfare and Ethical Review Body (AWERB) of the University of Glasgow and were accompanied by a UK Home Office project licence to the corresponding author (PE494BE48). Typically, ear punch samples were genotyped by Transnetyx. Arp3 floxed mice were obtained by *in vitro* fertilisation of frozen sperm from mice engineered to have floxed Arp3. C57Bl/6 mice were used for rederivation purposes. Arp3 transgene was detected by PCR using the primers (IL170) 5′-CAGCTATCCTGCATATCAGGTATGTACCT-3′, (IL173) 5′-AGGTGTTGACAACATGCTAAGAGCT-3′ and (IL174) 5′-AGGGACAGTCTGTGTTTCTCCTAATATC-3′. The oligonucleotides generate a 297-bp fragment for the wild-type (wt) allele and a 390-bp fragment for the floxed allele. Deletion was identified by a 600-bp fragment. PCR conditions were 30 s at 95°C, 30 s at 58°C and 30 s at 72°C for 34 cycles and a final extension at 72°C for 2 min. Tyrosinase Cre B (Tyr::Cre) mice in C57BL6/J background and their genotyping have been previously described (Delmas et al., 2003). Other alleles used for the purposes of this study have been previously described and are: DCT/*lacZ* (Mackenzie et al., 1997), Gt(ROSA)26Sor (Tyrosinase Cre-ERT2) (Hameyer et al., 2007), Cdkn2a (p16-Ink4a) (Krimpenfort et al., 2001) and Cdkn2a (p16/p19) (Serrano et al., 1996). Information about genotype and coat colour phenotype of cohort mice are provided in Table S1.

### Generation of primary mouse melanocyte cell lines and culture

Immortal melanocyte cell lines were generated as previously described (Li et al., 2011). Briefly, P2 pups derived from TyrCreER; Arp3^fl/fl^; Cdkn2a(p16/p19)^−/−^ mice were sacrificed and then the dorsal skin was dissected and cut into small pieces that were incubated in 1.5 ml solution of collagenases (type I, IV, Sigma-Aldrich) for 40 min at 37°C. Following centrifugation at 300 ***g***, pellets were resuspended in cell dissociation buffer (Gibco) and passed through 18 g and 20 g needles and strained through a 70 μm filter into 10 ml wash buffer (HBSS 10x Solution, Thermo Fisher Scientific, 14065056). Then pellets were resuspended in 4 ml of F-12 medium and cells were counted and plated into wells of a 6-well plate (1×10^6^ cells per well). Cells were allowed to settle for 4 days and then media were replaced with fresh media containing G418 (50 μg ml^−1^, Formedium, G418S). G418-containing medium was renewed every 4 days followed by extensive washes in PE buffer, [0.037% (w/v) EDTA in PBS] and pure melanocyte cultures were obtained 4-8 weeks following isolation. Cells were typically treated with DMSO or OHT (1 μM) for 7 days before experimental use, with media being renewed every 2 days. Cells were maintained in 10% foetal bovine serum, Primocin- (100 μg ml^−1^, InvivoGen, ant-pm-1) and PMA- (0.2 mM, Sigma-Aldrich, P1585) supplemented Ham's F-12 Nutrient Mix at 37°C and perfused with 5% CO_2_ and 21% O_2_. Pure melanocyte cultures were authenticated by validating the expression of established markers, including DCT, cKit and MITF. Cell lines were routinely tested for mycoplasma contamination.

### Immunohistochemistry of skin sections

Pup skin was shaved and dermal/epidermal skin layers were dissected from dorsal/ventral regions. Tissues were fixed in formalin overnight before embedding and formalin-fixed, paraffin-embedded (FFPE) tissues were cut into 4 μm sections using a Finesse Microtome ME+ (Thermo Scientific). The immunohistochemical staining was performed on an Agilent Autostainer link48. The sections underwent manual dewaxing through xylene, graded alcohol and were then washed in tap water before being loaded onto the autostainer. The sections then underwent peroxidase blocking (Agilent), and were also blocked in 2.5% normal horse serum before application of anti-S100 antibody for 40 min. Sections were then washed in Tris buffered Tween (Tbt; Thermo Scientific, TA-999-TT) before application of ImmPRESS-AP Polymer Anti-Rabbit IgG Reagent (Vector Laboratories, Rb-5401) for 30 min. Sections were rinsed in Tbt before applying ImmPACT Vector Red Alkaline Phosphatase Substrate (Vector Laboratories, SK-5105) for 15 min. The sections were then washed in water, counterstained with Haematoxylin and coverslipped using EcoMount. Stained sections were scanned and processed using the HALO Image Analysis Software (Indica Labs). Primary antibodies used are listed in Table S2.

### Immunofluorescence of skin or embryo tissue sections

FFPE embryos or skin samples were transversely sectioned, de-waxed in xylene, rehydrated through graded alcohols and then washed in tap water. Then, antigen retrieval was performed in citrate buffer (pH 6) for 25 min in a boiling water bath. Following, cooling at room temperature (RT) for 30 min, sections were then blocked with 5% normal donkey serum in NDS/PBS (Sigma-Aldrich, SLBQ9773L) for 30 min at RT and incubated overnight with primary antibody in 5% NDS/PBS at 4°C. Following washing, sections were incubated with fluorophore-conjugated secondary antibody in 5% NDS/PBS for 60 min at RT and subsequently with DAPI for 5 min at RT. Sections were then washed and mounted with Prolong Diamond Antifade reagent (Invitrogen, P36965). Primary and secondary antibodies used are listed in Table S2. Images were acquired using a Zeiss 710 confocal microscope equipped with an Ec Plan Neofluar 20×/0.50 dry lens and an Ec Plan Neofluar 40×/1.30 oil objective.

### Proliferation (BrdU) assessment of melanoblasts

Melanoblast proliferation was assessed at E13.5. Mothers were injected with BrdU (BD Bioscience) for 2 h before embryo harvesting. Embryos were fixed in 4% paraformaldehyde (PFA), cut transversely and embedded back-to-back into paraffin blocks. Then 5 μm sections were made on three depth levels within the trunk of each embryo, each level being 75 μm apart. Sections from all levels were stained fluorescently as described above to visualise DCT, BrdU and collagen IV expression within the skin. BrdU-labelled nuclei and dermal/epidermal (melanoblasts above or below the basement membrane) ratios were quantified.

### Cleaved caspase 3 staining of melanoblasts

Melanoblast apoptosis was assessed at E13.5. Embryos were fixed in 4% PFA, cut transversely and embedded back-to-back into paraffin blocks. Then 5 μm sections were made on three depth levels within each embryo, each level being 75 μm apart. Sections from all levels were stained fluorescently as described above to visualise DCT and cleaved caspase 3 expression within the skin. To validate cleaved caspase 3 staining, melanocytes either treated with DMSO or with a combination of the Bcl2 and Bcl-xL inhibitor ABT-737 (10 μΜ; Selleckchem, S1002) and the mRNA transcription inhibitor Actinomycin D (1 μM; Sigma-Aldrich, A9415) for 6 h and were then embedded in agarose, fixed in 10% neutral buffered formalin at 4°C for 16 h and embedded in paraffin for sectioning as described above.

### β-Galactosidase assay

Embryos were harvested from timed matings of Tyr::Cre; Arp3^wt/fl^; DCT/*lacZ*^+^ mice at E11.5, E13.5 and E15.5 and were stained as previously described (Ma et al., 2013). Embryos were typically harvested in PBS and fixed immediately in ice-cold 0.25% glutaraldehyde in PBS for 30 min at 4°C. Following washing in ice-cold PBS for 10 min, embryos were placed in permeabilization buffer (2 mM MgCl_2_, 0.01% sodium deoxycholate and 0.02% NP-40) for 30 min at RT. Embryos were then incubated with X-gal staining buffer [2 mM MgCl_2_, 0.01% sodium deoxycholate, 0.02% NP-40, 5 mM K_4_Fe(CN)_6_, 5 mM K_3_Fe(CN)_6_, 1 mg ml^−1^ X-gal (Promega)] for 48 h at 4°C. Embryos were then washed extensively with PBS and images were acquired using a Zeiss Stemi-2000 dissection microscope equipped with a Canon DS126191 EOS camera (EOS Utility, Edmund Optics). For melanoblast quantification in the trunks, an area of 876×1248 pixels^2^ was cropped and gridded with an area per point of 36,700 pixels^2^. For melanoblast quantification in the forelimb, an area of 1008×2664 pixels^2^ was cropped to cover most forelimb skin and gridded with an area per point of 120,000 pixels^2^. For melanoblast quantification in the back, an area of 1002×258 pixels^2^ was cropped and gridded with an area per point of 29,000 pixels^2^. For melanoblast quantification in the head, an area of 522×588 pixels^2^ was cropped next to the eye and gridded with an area per point of 16,900 pixels^2^. Cell number in subareas was counted for E11.5, E13.5 and E15.5 comparing wild-type, Arp3^+/−^ and Arp3^+/+^ embryos. All quantifications were in Fiji software (ImageJ v2.0.0) and all plots were generated using Prism 8 (v8.2.0; GraphPad Software).

### Immunoblotting

DMSO- and OHT-treated melanocytes were lysed in RIPA buffer (50 mM Tris-HCl, 150 mM NaCl, 1% NP-40 and 0.25% Na-deoxycholate) with protease and phosphatase inhibitor cocktail (Thermo Fisher Scientific). Lysates were centrifuged at 15,000 rpm (22,640 ***g***) for 10 min and typically 20 μg of protein were separated by SDS-PAGE electrophoresis. Proteins were then transferred to PVDF membranes, which were subsequently incubated with primary antibody for 16 h at 4°C. Protein detection was achieved through a 1 h incubation with Alexa Fluor-conjugated secondary antibodies. Signal was imaged using the automated signal intensity mode of the Li-Cor Odyssey CLx system. All images were processed and analysed using the Li-Cor Image Studio Lite software, version 5.2.5. Primary and secondary antibodies used are listed in Table S2.

### Growth curves of melanocytes

DMSO- or OHT-treated melanocytes were plated in 12-well plates in triplicate wells (5×10^4^ cells per well) and allowed to adhere overnight. Cells were counted after 24, 48 and 96 h using a CASY Model TT Cell Counter (Innovartis, Roche Applied Science). Media were renewed every 48 h.

### DNA extraction from melanocytes and PCR

DNA was extracted from DMSO- or OHT-treated melanocytes using the QIAamp DNA Mini kit (Qiagen) according to the manufacturer's instructions. PCR was performed with primers IL170, IL173 and IL174 as described above. PCR conditions were 30 s at 95°C, 30 s at 58°C and 30 s at 72°C for 34 cycles and a final extension at 72°C for 2 min.

### qRT-PCR

RNA was isolated from DMSO- or OHT-treated melanocytes using the RNeasy Mini Kit (Qiagen) according to the manufacturer's instructions and was used for cDNA synthesis with DyNAmo cDNA synthesis kit (F-470L, Thermo Fisher Scientific). Then qRT-PCR was performed using the DyNAmo HS SYBR Green qPCR kit (F410L, Thermo Fisher Scientific). Real-time PCR was performed on a QuantStudio 3 Real-Time PCR System (Applied Biosystems, Thermo Fisher Scientific) as follows: 3 min at 95°C, 40-cycles of 20 s at 95°C, 30 s at 57°C, 30 s at 72°C and a final 5 min at 72°C. Relative mRNA quantification was performed using the 2^−ddCT^ method for multiple genes. Primer sequences are provided in Table S3.

### Live cell imaging and random migration assay

DMSO- or OHT-treated melanocytes were plated at low confluency onto fibronectin-coated dishes and imaged for 16 h with acquisitions at 20 min intervals using a Nikon TE2000 microscope equipped with a Plan Fluor 10×/0.30 objective and a heated CO_2_ chamber. Images were analysed with Fiji software (ImageJ v2.0.0).

### Polyacrylamide hydrogel preparation

Polyacrylamide hydrogels were synthesized to 0.7 kPa, 7 kPa and 38 kPa stiffness values as previously described (Papalazarou et al., 2020). For functionalization with ECM, polyacrylamide hydrogels were treated with 0.2 mg ml^−1^ sulfo-SANPAH solution in MilliQ water (Thermo Fisher Scientific, 22589) followed by UV irradiation (365 nm) for 10 min. Hydrogels were extensively washed with 50 mM HEPES buffer (pH 8.5), incubated overnight with fibronectin (10 μg ml^−1^) and washed extensively in PBS before use.

### Immunofluorescence

DMSO- or OHT-treated melanocytes (10^4^ cells per cm^2^) were plated on fibronectin-coated 19-mm diameter coverslips or PAAm hydrogels. Cells were fixed in 4% PFA for 10 min and then permeabilised with 0.1% Triton X-100 for 5 min followed by 30 min incubation in 1% bovine serum albumin. Following incubation with primary antibody for 60 min, cells were washed and detection was performed using fluorescently-labelled secondary antibodies. Nuclei were visualised with DAPI (0.5 μg ml^−1^; D1306, Invitrogen) and F-actin with Alexa Fluor 647 Phalloidin (1:100 dilution; A22287, Invitrogen). Coverslips were mounted using ProLong Diamond antifade reagent (P36965, Invitrogen). Images were acquired using a Zeiss 880 Laser Scanning Microscope with Airyscan equipped with a Plan-Apochromat 63×/1.4 oil DIC M27 objective or a Nikon A1R confocal microscope equipped with Nikon Plan Fluor 20×/1.30 objective (Fig. S5I). Primary and secondary antibodies used are listed in Table S2.

### Quantifications

Melanoblast length in Fig. 3A was quantified manually using Fiji software (ImageJ v2.0.0) using the line tool to measure the length (longest axis) of randomly selected *lacZ*-positive melanoblasts. Cell shape analysis in Fig. 5B,C was performed by manually drawing around the cell perimeter based on F-actin staining and measuring the area and the shape descriptors using the Fiji software (ImageJ v2.0.0). Focal adhesion analysis in Fig. 5E,F and Fig. S5C,D,F,G was performed using the Fiji software (ImageJ v2.0.0), applying an automated threshold and the ‘analyze particles’ function with a minimum particle area threshold of 0.15 μm^2^. Cell shape analysis in Fig. 5K was performed using Cell Profiler software (v3.0.0; CellProfiler), applying a mask for cell area based on F-actin staining. Raw data associated with each figure can be found in Table S4.

### Statistics and reproducibility

All datasets were analysed and plotted using Prism 8 (v8.4.3; GraphPad Software) unless otherwise stated. Differences between groups were tested for normal distribution and analysed using the appropriate statistical test, as mentioned in each figure legend. Error bars represent s.d., unless otherwise stated. No statistical method was used to predetermine sample size. Sample sizes were chosen according to usual practice for each experimental design based on the host's lab experience and pilot experiments to estimate variability. No data were excluded from analysis. Regarding *in vitro* experiments, unless otherwise stated, cells from the same passage were randomly treated and analysed during each experiment. At least three different passages were used for biological repeats unless otherwise stated. The investigator was not blinded during group allocation or outcome assessment. Where applicable, we performed automated software analyses to reduce chance of bias.

## Supplementary Material

Supplementary information
