# Supplementary material for: The Arp2/3 complex is crucial for colonisation of the mouse skin by melanoblasts
Source: Development. 2020 Nov 15;147(22):dev194555. doi: 10.1242/dev.194555 (PMC7687863; doi:10.1242/dev.194555)
Supplement: Supplementary information [file develop-147-194555-s1.pdf]

Figure S1

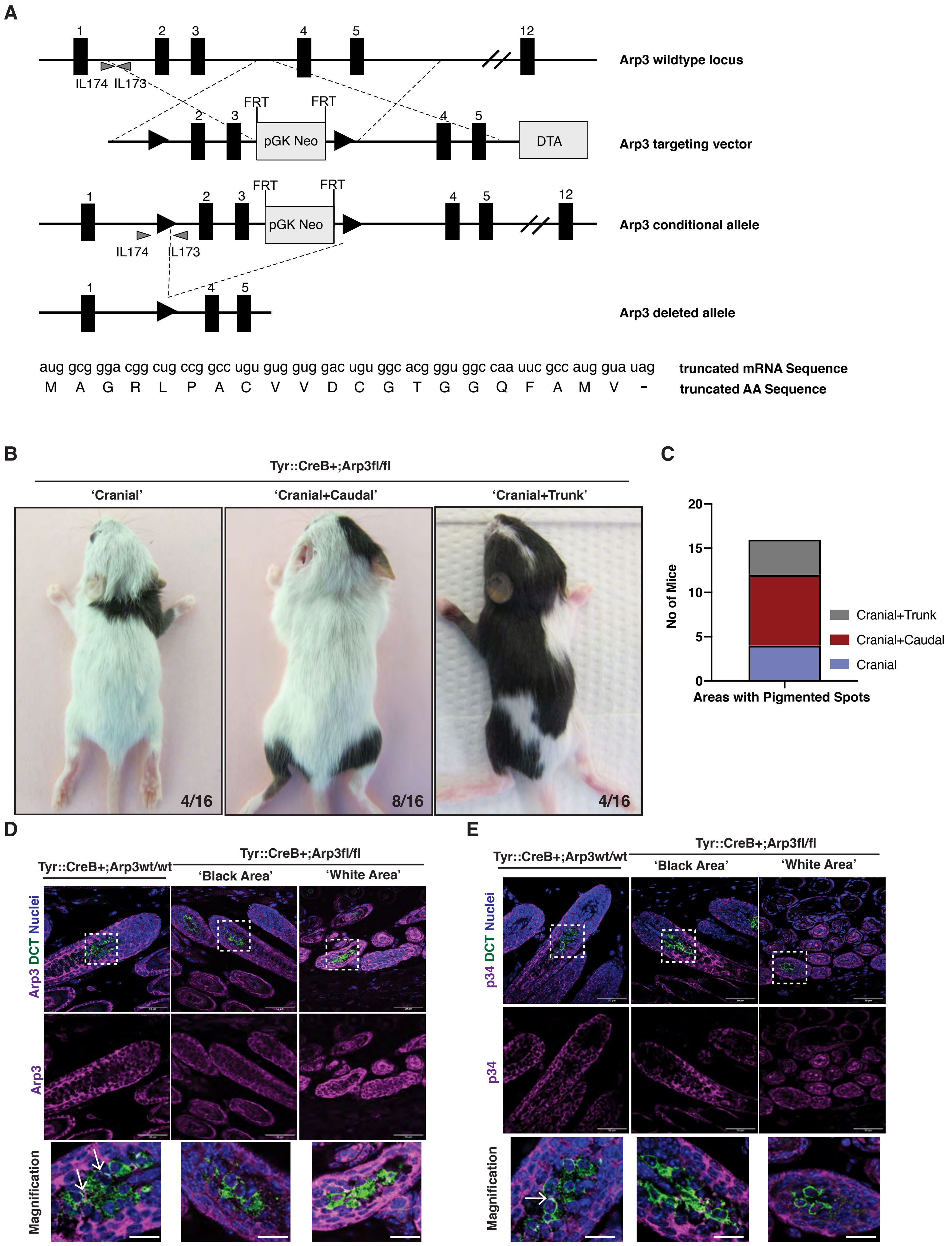

Development • Supplementary information

Figure S1 – Arp3 f/f Tyr::Cre+ mice show reduced number of hair follicle melanocytes.

(A) Schematic representation of generation of the conditional Arp3loxp/loxp mouse allele. The pGK-Neomycin (neo) and diphtheria toxin (DTA) cassettes as well as loxP sites were introduced *via* recombination in bacteria into the Arp3 wild-type gene as shown. The resulting allele allows for double selection and deletion of exons 2 and 3 by Cre recombinase. The predicted mRNA and amino acid sequences after cre-mediated excision of exon 2 and 3 are shown. Grey arrows indicate position of primers IL173 and IL174 used to genotype the conditional Arp3 allele.

(B) Representative examples of dorsal coat colour variations of Arp3 fl/fl Tyr::Cre+ mice showing pigmented spots in cranial, caudal and trunk areas.

(C) Number of mice per phenotype from (A). n = 4 for 'Cranial', n = 8 for 'Cranial and Caudal' and n = 4 for Cranial and Trunk pigmentation.

(D) Top; Immunofluorescence of dorsal skin sections of P14 control as well as 'black' and 'white' patches from Arp3 fl/fl Tyr::Cre+ mice showing Arp3 (magenta), DCT (green) and nuclei (blue). Middle; Arp3 channel from top. Bottom; Magnification of areas indicated by a white box. Scale bars, 50 µm.

(E) Top; Immunofluorescence of dorsal skin sections of P14 control as well as 'black' and 'white' patches from Arp3 fl/fl Tyr::Cre+ mice showing p34 (magenta), DCT (green) and nuclei (blue). Middle; p34 channel from top. Bottom; Magnification of areas indicated by a white box. Scale bars, 50 µm.

Development • Supplementary information

Figure S2

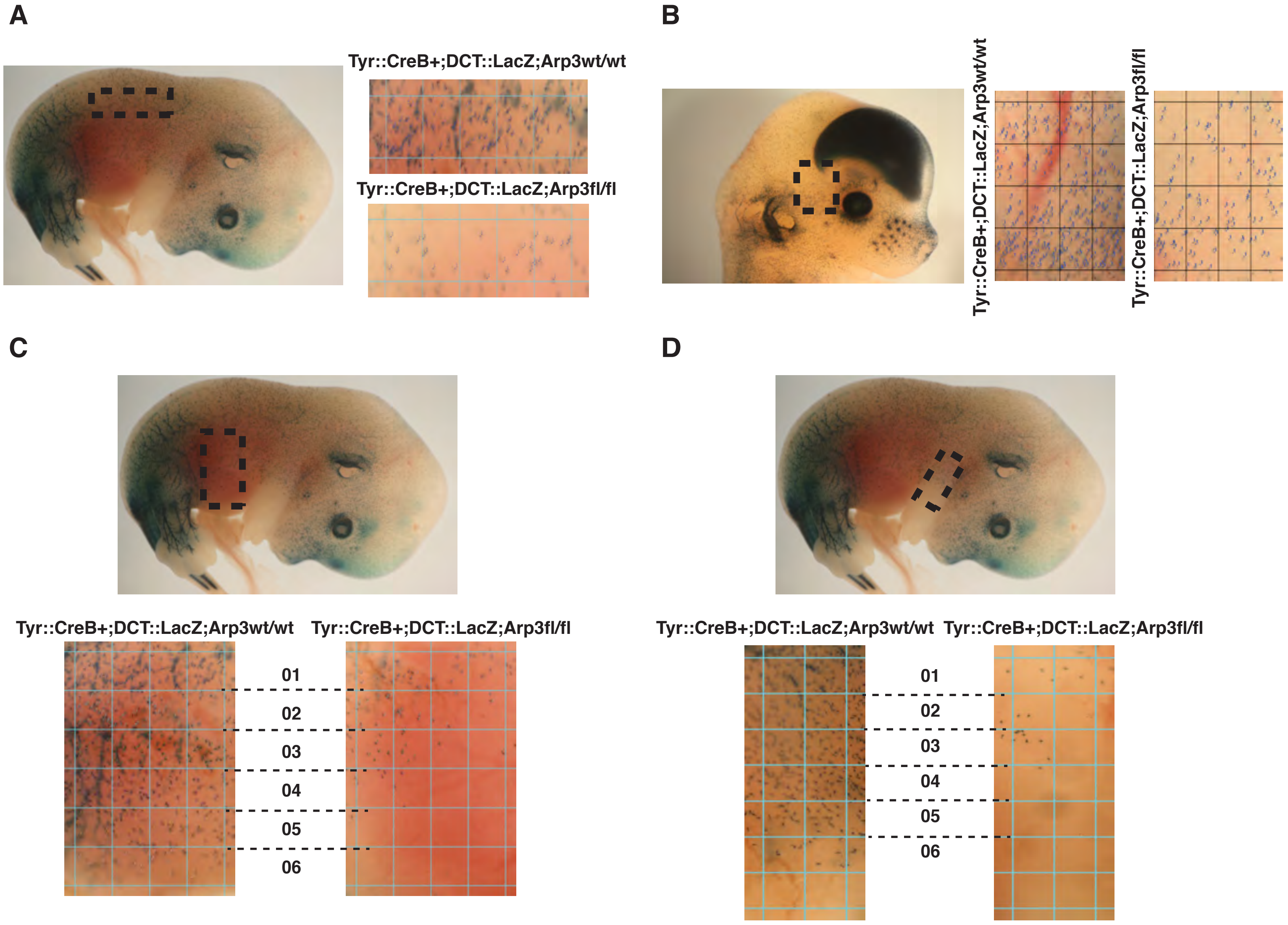

Figure S2 – Quantification of melanoblast number and distribution in embryos.

(A - D) Embryos are DCT::LacZ; Tyr::Cre$_{+}$; Arp3 wt/wt. Black dotted boxes indicate regions for quantification regarding analysis of trunk (A), head (B), belly (C) and forelimb (D) regions. For (C) and (D) six equally spaced regions were assigned and number of melanoblasts per region was calculated to assess the migratory front of melanoblasts for each embryo.

Figure S3

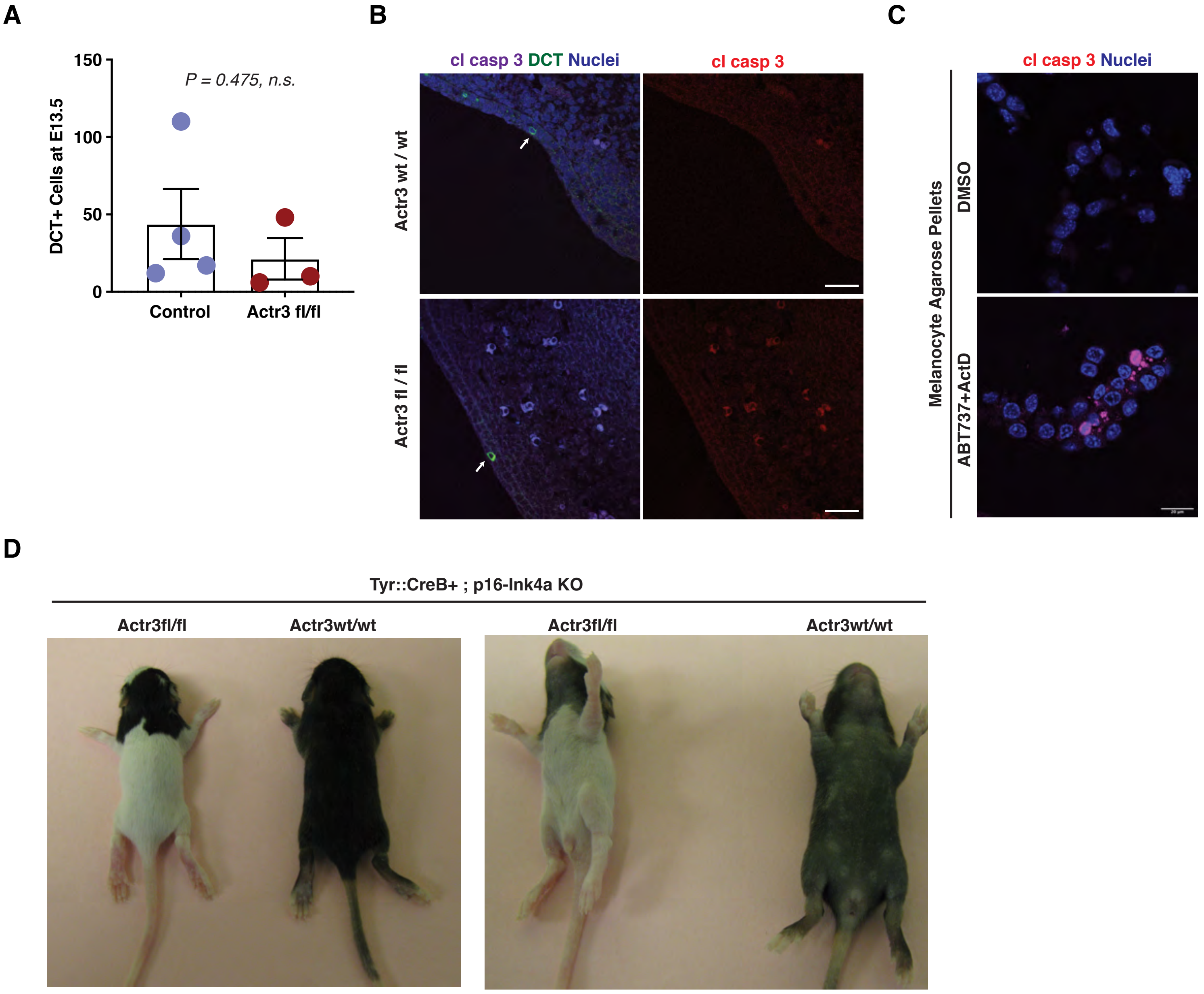
A
B
C
D
150
100
50
0
DCT+ Cells at E13.5
P = 0.475, n.s.
Control
Actr3 fl/fl
cl casp 3 DCT Nuclei
cl casp 3
Actr3 wt / wt
Actr3 fl / fl
cl casp 3 Nuclei
Melanocyte Agarose Pellets
DMSO
ABT737+ActD
Tyr::CreB+ ; p16-Ink4a KO
Actr3fl/fl
Actr3wt/wt
Actr3fl/fl
Actr3wt/wt

Figure S3 – Loss of Arp3 didn't induce cleaved-caspase 3 expression in melanoblasts at E13.5 and Arp3 f/f Tyr::Cre$_+$:p16-Ink4a$_{-/-}$ mice display coat pigmentation defects.

(A) Number of DCT positive cells in E13.5 DCT::LacZ; Tyr::Cre$_+$ Arp3 wt/wt and Arp3 fl/fl embryo transverse sections. Each dot represents an embryo. Values are mean ± SEM of n = 4 wt/wt and n = 3 fl/fl embryos from 4 litters. Statistical significance was assessed by two-tailed, unpaired t-test.

(B) Representative images of E13.5 DCT::LacZ; Tyr::Cre$_+$ Arp3 wt/wt (top) and Arp3 fl/fl (bottom) embryo transverse sections showing cleaved caspase 3 (magenta), DCT (green) and nuclei (blue). Scale bars, 50 μm. White arrows indicate melanoblasts (DCT-positive). Right panel; cleaved caspase 3 channel (red). Pictures representative from n=3 embryos per condition.

(C) Representative images of sections of melanocytes in FFPE agar plugs stained by IHC against cleaved caspase 3 (red) and DAPI (blue). Melanocytes have been treated before embedding either with DMSO (left) or with a combination of the Bcl-2 and Bcl-xL inhibitor ABT-737 (10 μM) and the mRNA transcription inhibitor Actinomycin D (1 μM) for 6 hours to induce apoptotic cell death (right).

(D) Top; Coat colour of P12 Arp3 f/f Tyr::Cre$_+$ mice with control (Arp3 wt/wt) littermate.

Development • Supplementary information

Figure S4

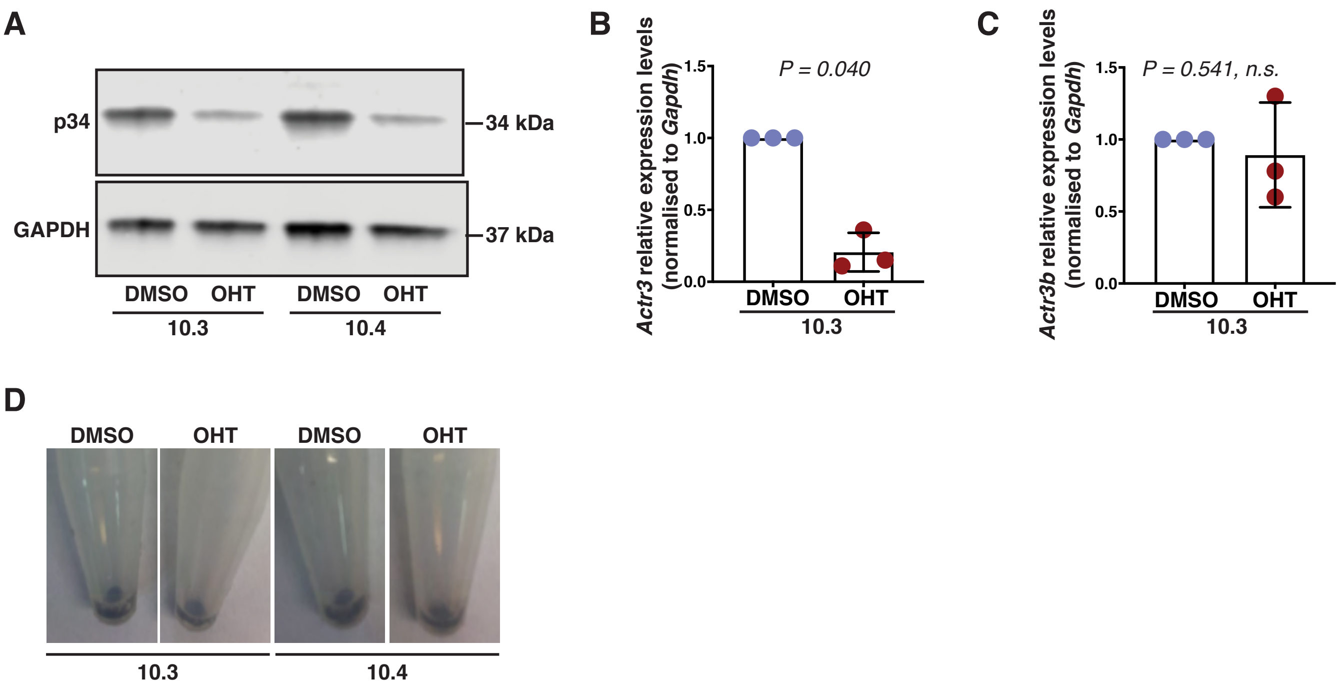

Figure S4 – Arp3 depletion in primary murine melanocytes.

(A) Immortalised primary melanocyte 10.3 and 10.4 lines were treated with OHT or DMSO for 7 days and immunoblotted for p34 and GAPDH (loading control). Pictures are representative for 3 independent experiments.

(B-C) 10.3 immortalised primary melanocytes were treated with OHT or DMSO for 7 days and *Arp3* (B) and *Arp3b* (C) expression was measured with qRT-PCR. Values are mean ± SD and relative to control from 3 independent experiments. Statistical significance was assessed by two-tailed, one-sample t-test on natural log-transformed values.

(D) Cell pellets of DMSO- or OHT-treated 10.3 and 10.4 primary melanocytes in PBS.

Development • Supplementary information

Figure S5

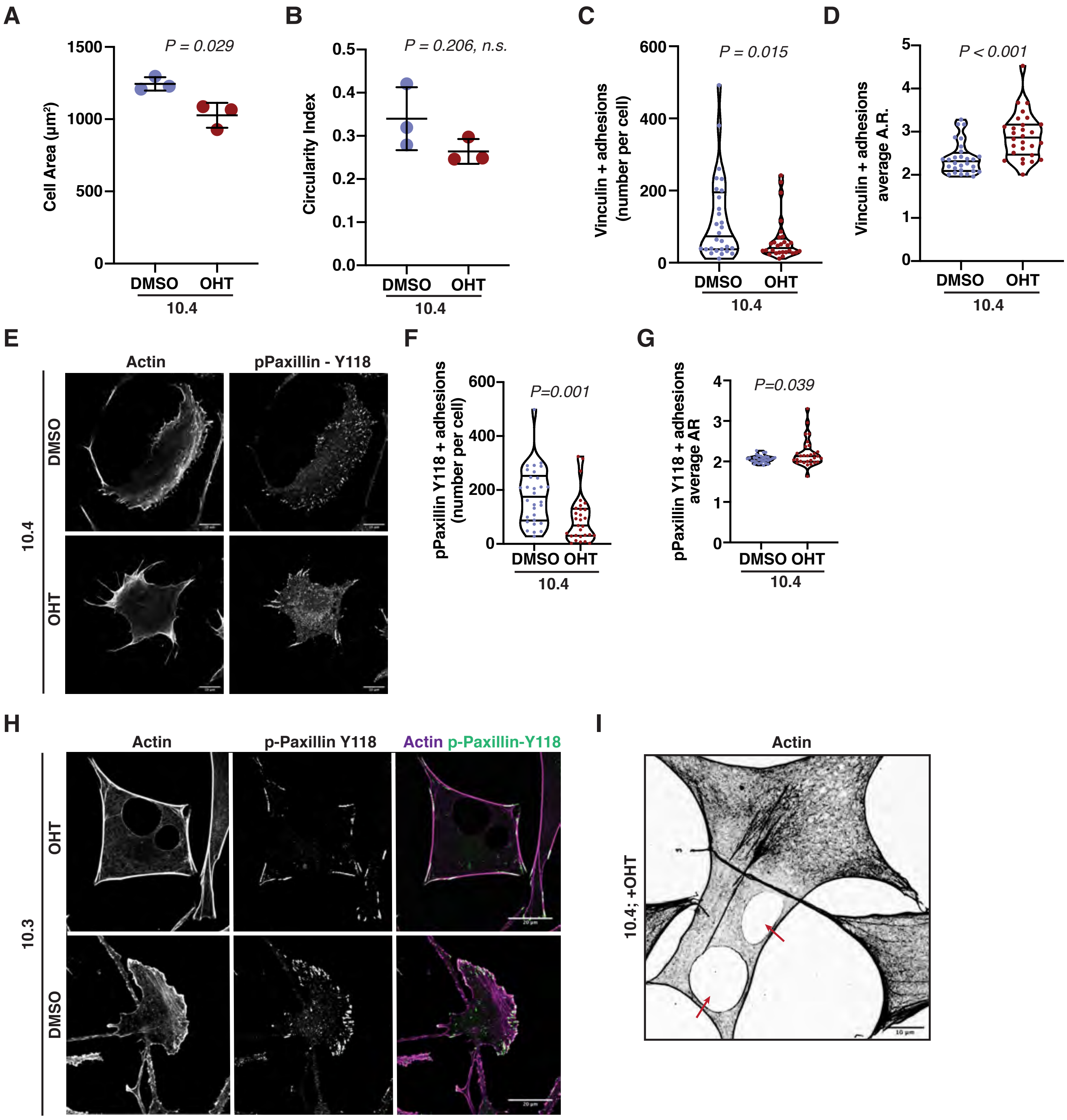

Development • Supplementary information

Figure S5 – Arp3 f/f melanocytes show shape changes, decreased focal adhesions and cell body holes.

(A-B) Quantification of cell shape in DMSO- or OHT-treated 10.4 primary melanocytes including cell area (μm2) (A) and circularity index (B). Values are mean per experiment ±SD from 3 independent experiments. Welch's t test.

(C-D) Quantification of Vinculin-positive particles in DMSO- or OHT-treated 10.4 primary melanocytes showing the number of focal adhesions per cell (C) and the average aspect ratio (A.R.) of focal adhesions per cell (D). Values are median and interquartile range from n = 28 DMSO and n = 28 OHT-treated cells. Cells are from three independent experiments. Mann-Whitney U test.

(E) Immunofluorescence of 10.4 immortalised primary melanocytes treated with DMSO or OHT for 7 days and plated on fibronectin-coated coverslips showing Actin (left) and pPaxillin-Y118 (right). Scale bars, 10 μm.

(F-G) Quantification of p-Paxillin-Y118-positive particles in (E) showing the number of focal adhesions per cell (F) and the average aspect ratio (A.R.) of focal adhesions per cell (G). Values are median and interquartile range from n = 30 DMSO- and n = 29 OHT-treated cells. Cells are from three independent experiments. Welch's t test.

(H) Immunofluorescence of 10.3 immortalised, primary melanocytes treated with DMSO or OHT for 7 days and plated on fibronectin-coated coverslips showing Actin (left) and pPaxillin-Y118 (middle). Right; Merged images of Actin (magenta) and p-Paxillin (Y118) (green). Scale bars, 10 μm.

(I) Immunofluorescence of OHT-treated melanocytes from (G) showing Actin. Red arrows indicate cell body holes. Scale bar, 10 μm.

Development • Supplementary information

Table S1. Mouse cohort data

Click here to Download Table S1

Table S2. Antibody reagents

Click here to Download Table S2

Table S3. RNA silencing reagents

Click here to Download Table S3

Table S4. Source data

Click here to Download Table S4

Development • Supplementary information
